# Biological Treatments in Atopic Dermatitis

**DOI:** 10.3390/jcm4040593

**Published:** 2015-04-03

**Authors:** Andrea Montes-Torres, Mar Llamas-Velasco, Alejandra Pérez-Plaza, Guillermo Solano-López, Javier Sánchez-Pérez

**Affiliations:** Department of Dermatology, Hospital Universitario de La Princesa, Diego de León 62, Madrid 28006, Spain; E-Mails: mar.llamasvelasco@gmail.com (M.L.-V.); aperezplaza@gmail.com (A.P.-P.); guitje1@hotmail.com (G.S.-L.); jsanchezperez@aedv.es (J.S.-P.)

**Keywords:** atopic eczema, atopic dermatitis, pathogenesis, biologics, antibody therapy

## Abstract

Atopic dermatitis (AD) is one of the most common chronic inflammatory skin diseases that affect both children and adults with a prevalence of 30% and 10%, respectively. Even though most of patients respond satisfactory to topical anti-inflammatory drugs, about 10% require one or more systemic treatments to achieve good control of their illness. The progressive and increasingly detailed knowledge in the immunopathogenesis of AD has allowed research on new therapeutic targets with very promising results in the field of biological therapy. In this article, we will review the different biological treatments with a focus on novel drugs. Their mechanism of action, current status and results from clinical trials and observational studies will be specified.

## 1. Introduction

Atopic dermatitis (AD) is a pruritic and chronic inflammatory dermatosis with a high prevalence in industrialized countries. AD frequency varies between 7% to 30% of children and 1% to 10% of adults entailing an important decline of their quality of life [1,2,3,4,5,6]. Up to 60% of the cases of AD begin within the first year of life and up to 95% start before the age of five [7,8]. AD manifests through outburst with an interindividual variability in length but presents a progressively improving tendency, thus, three out of four cases show a spontaneous remission after puberty [1,7]. Although etiopathogenic factors are not completely understood, immunologic, genetic and environmental factors are interrelated to produce a skin barrier disturbance as well as an immunologic dysregulation. Filaggrin gene mutation as well as a characteristically biphasic pattern involving T helper type 2 (Th2) and Th1 cells along the acute and chronic phase of AD are the traditionally key factors in AD pathogeny [3,6,7,9,10]. The involvement of Th17 and Th22 cells in AD pathogeny has been more recently published [11,12]. There are many therapeutic possibilities to temporarily control signs and symptoms but none of them is able to cure the disease. Thus, outbreaks after treatment suspension are a frequent fact. Most of the cases can be controlled with topical corticosteroids or topical calcineurin inhibitors, but moderate to severe AD cases sometimes require the addition of a systemic treatment to achieve enough control. Cyclosporine, methotrexate, azathioprine, mycophenolate mofetil are established alternatives when systemic treatment is required [3,9,13,14]. Nevertheless, interindividual response variability and these drugs’ known secondary adverse effects are the rationale to look for new drugs that achieve a better control of the disease while decreasing secondary adverse effect risk [3,14,15]. Advances in AD etiopathogenic knowledge allow the identification of further targets for biological treatments as therapeutic alternative treatments *vs.* systemic treatments [3,5,6,15,16,17].

We have conducted a thorough review on the different therapeutic possibilities regarding the use of biologic agents in AD with special focus on novel treatments. Two different databases, clinicaltrials.org and pubmed.com, have been consulted with last access on 31 December 2014. The following keywords: “(atopic dermatitis OR atopic eczema) AND (biologics OR biological treatment OR antibody treatment)” were initially used, obtaining a large number of papers. First, we selected those articles published within the last five years, in Spanish or English language and discussing biological therapy of AD in the abstract or when it is suggested in the article tittle. More than 40 clinical trials, observational studies, case reports and reviews of novel therapies were found and in-depth reviewed. In addition, relevant references cited in these articles have been also reviewed in detail. Simultaneously, a search using the keyword “atopic dermatitis” in clinicaltrials.gov was made. Of a total of 437 clinical trials performed about AD, we only included those in which biological drugs were tested and clinical parameters were measured. Finally, a new search in pubmed.com about mechanisms of action and roles in AD pathogeny was performed for each drug previously included. Based on this data, we then listed each drug in alphabetical order, their mechanism of action and involvement in the pathogenesis of AD as well as a description and most of the clinically relevant results of the different studies published to date.

## 2. Biologics in Atopic Dermatitis

### 2.1. CD20 Directed Therapy

#### Rituximab

Rituximab is a chimeric monoclonal antibody against CD20, an antigen that is present on the surface of B cells [12,18,19]. Such interaction can cause B cell lysis by antibody-dependent cellular cytotoxicity, complement-dependent toxicity or apoptosis [3,5,18]. The result is a B lymphocyte depletion and, therefore, an inhibitory effect on various immunological mechanisms as antibodies production or T-dependent B cell activation due to its function as antigen presenting cells [18,20,21]. Its efficacy in the treatment of several autoimmune diseases has been shown [18,20] and some authors have already tried to evaluate its effectiveness in patients with severe AD refractory to other systemic therapies.

Simon *et al.* reported their experience after treating six patients with severe AD with rituximab (Table 1). At week 4, all patients showed a significant improvement in skin lesions and pruritus with a significant decrease, up to 70%, in the Eczema Area and Severity Index (EASI) score. Such clinical improvement was maintained for at least 24 weeks in five of the six patients. The number of CD20+ B lymphocytes in peripheral blood was undetectable after the third day of treatment, whereas only a decrease of 50% was observed in affected skin after treatment with rituximab. Regarding the total immunoglobulin E (IgE) levels, contrary to what one would expect, only a slight decrease was observed after treatment [22]. Significant improvement was also observed in a woman with severe AD after receiving a single dose of 1000 mg rituximab (second dose was suspended because of pregnancy). The total body surface area decreased from 90% to 5%, and the improvement was maintained for 17 months. No side effects were reported regarding pregnancy [23]. However, opposite results were observed in two patients with severe AD. The treatment with two infusions of rituximab 500 mg given two weeks apart did not achieve a significant improvement. Regarding IgE levels, no variation was observed after treatment [24].

The fact that IgE levels remain practically unchanged during the treatment with rituximab is due to the lack of CD20 antigen on the surface of plasma cells. These cells escape from the action of rituximab and continue producing immunoglobulins [22]. Longer therapies and/or longer follow-up periods may possibly be required for the B lymphocyte depletion to have an impact on IgE production. On the other hand, other rituximab mechanisms of action have begun to gain importance. Probably, the loss of B cells and of its function as antigen presenting cells will entail a lower T cell activation and thus a lower cytokine and mediator release. This mechanism may probably be responsible for the clinical improvement in these patients [22,24].

### 2.2. IgE Directed Therapy

#### 2.2.1. Omalizumab

Omalizumab is a humanized monoclonal antibody directed selectively against circulating IgE. By blocking IgE, it prevents its interaction from its high affinity receptor (FcεRI), normally present in the membrane of basophils and mast cells. Thereby, omalizumab inhibits the activation, degranulation and release of distinct mediators involved in the pathogenesis of AD [5,19,25,26,27]. More recently, the omalizumab inhibitory role on FcεRI expression on the surface of dendritic cells, whose expression is increased in atopic patients [27], has been described [28]. In this sense, omalizumab also inhibits antigen presentation by the FcεRI-IgE complex (dendritic cell) to T lymphocytes [27]. Its use is currently approved for the treatment of asthma and chronic spontaneous urticaria in patients aged older than 6 and 12 years, respectively [29]. In the subcutaneous administration, the dose is adjusted depending on weight and baseline IgE levels [25].

Since 80% of patients with AD exhibit elevated levels of IgE [27], efforts to extrapolate its use to patients with refractory AD have been made. However, the results have been highly variable, with both positive and negative results in patients with normal or elevated IgE levels.

Twenty-one patients with AD and moderate-severe persistent asthma were evaluated in one of the first studies of omalizumab in this field (Table 1). A clinically and statistically significant improvement in all cases was observed. This improvement was even more noteworthy in the group of patients with normal pretreatment IgE levels [30]. Positive but somewhat less favorable results were subsequently obtained in a study of 10 subjects with AD treated with omalizumab for severe persistent asthma (Table 1). A scoring atopic dermatitis (SCORAD) average decrease of 25% was observed. Seven of the ten patients achieved a good (SCORAD reduction of 25%–50%) or very good response (>50%) two months after finishing treatment [31]. An important clinical improvement was also evident in three patients aged 10–13 years, with recalcitrant AD (Table 1). All three patients had strikingly increased serum IgE levels. Side effects were not observed in this group [32]. A few years ago, Fernández Martínez-Antón *et al.* also published their experience in nine patients with severe AD refractory to at least two systemic treatments (Table 1). All of them underwent improvement regarding itching and quality of life, although, only two patients achieved good control of eczema [25]. Similar results were also obtained in a study of 11 patients with AD (Table 1). The doses used were noteworthy and significantly lower compared to other previously published papers [33]. In a subsequent study of 20 patients with severe AD, no statistically significant difference between the omalizumab and placebo group treated for 16 weeks was observed (Table 1). The presence of long-standing AD, unlike other studies, could explain such discrepancies according to them [34]. No clinical response was obtained in a series of three patients with severe AD either (Table 1). All three patients presented high serum IgE levels and thus, it is possible that the given omalizumab dose was not enough [35]. Very similar results were observed in another study controlled with placebo (Table 1). Despite a significant decrease in IgE levels in omalizumab group, no statistically significant differences could be observed from the cutaneous point of view [36]. A new phase IV study in order to assess the efficacy of omalizumab in the management of AD in childhood will begin shortly. It is estimated that a total of 62 patients aged 4–19 years will have been recruited to be randomly assigned placebo or omalizumab. Dose is still unspecified (NCT02300701) [36].

Despite several promising findings, there is a great variability of results in the AD field. Theoretically, a poorer response to omalizumab in patients with higher levels of IgE could have been expected, due to dosing adjustment and the impossibility of overtaking the maximum tolerated dose. However, favorable results were obtained in both patients with normal and elevated IgE levels [30,32]. Even within the same case series, the patients with better clinical response presented higher IgE levels [33]. Moreover, the variability of the doses used, duration and different clinical severity may explain these results; on the other hand, the absence of clinical improvement while decreasing serum IgE levels could be justified by the existence of many other etiopathogenic factors [37].

#### 2.2.2. Ligelizumab

A novel humanized monoclonal antibody against IgE called ligelizumab has been recently developed. Its efficacy and safety in patients with moderate-severe AD have been evaluated in a phase II, randomized, controlled, double blind (RCDB) study (Table 1). No study results have been published so far (NCT01552629) [38].

### 2.3. IL-1 Directed Therapy

Anakinra is a recombinant human receptor antagonist against interleukin (IL)-1, a pro-inflammatory cytokine involved in the pathogenesis of various autoimmune and chronic inflammatory diseases. Anakinra blocks the biological activity of both isoforms, IL-1α and β, by competitively inhibiting the binding to their receptor, which seems to be involved in the initiation and maintenance of Th2 response [39,40,41,42]. We could not find any study on anakinra efficacy in patients with severe AD. Recently, a phase I pilot study was carried out to evaluate the efficacy and safety of anakinra in young patients with severe AD. However, the recruitment process has been suspended with only one patient recruited (NCT01122914) [43].

### 2.4. IL-4 Directed Therapy

#### 2.4.1. Dupilumab

IL-4 is the cytokine responsible for promoting Th2 cell differentiation and consequently the secondary production of IL-4 and IL-13, potent stimulators of IgE production by B lymphocytes [5,44]. The presence of a predominant Th2 cell phenotypic response in the acute phase of AD is well known. Recently, Gittler *et al.* have demonstrated a significant increase in gene expression of IL-4, IL-13 and IL-31 in biopsies of acute lesions in 10 patients with AD [12].

Dupilumab is a fully human monoclonal antibody directed against the alpha subunit of IL-4 receptor. Given that such subunit is also shared with IL-13 receptor, both pathways will be inhibited [45,46,47]. It currently constitutes one of the biggest therapeutic promises in the AD management and at this moment, it is in phase III clinical trial for such indication.

Previous phase I and II studies already showed very promising results with an acceptable safety profile. These studies consist of four RCDB trials in which the use of dupilumab alone or associated with topical steroid treatment was evaluated (Table 1). In the four-week monotherapy studies, the intake of dupilumab was associated with rapid and dose-dependent improvements in the EASI score, the investigator’s global assessment score and pruritus. This improvement was further increased after 12 weeks of continuous treatment with 300 mg of dupilumab. A 50% or greater reduction in the EASI score was achieved in the 85% of patients. A significant decrease was also observed regarding pruritus. In the four-week combination therapy, the 100% of dupilumab group reached a significant clinical improvement. It should be noted that the dupilumab treatment in monotherapy and in combination therapy did not result in an increased incidence of side effects compared to placebo. Dupilumab group showed a higher incidence of injection site reaction, headache and nasopharyngitis than placebo group, although a similar frequency of adverse events was observed in both groups [48].

Three new phase III trials are currently being developed. The first one, an open label study, evaluates the long-term safety and efficacy of repeat doses of dupilumab in adults who had previously participated in dupilumab controlled studies (NCT01949311) [49]. The remaining two RCDB studies evaluate the efficacy and safety of using dupilumab, either alone or in combination with topical corticosteroids, in patients with severe-moderate AD. An estimated number of 600 and 700 patients will have been recruited in each study, respectively (NCT02277769 and NCT02260986) [50,51].

#### 2.4.2. Pitrakinra

Pitrakinra is a recombinant human IL-4 protein capable of specifically binding to the alpha subunit of the IL-4 receptor. Like dupilumab, it inhibits the downstream signaling pathways of both IL-4 and IL-13 [52,53]. The first trial in patients with AD, a RCDB study, has been already completed (Table 1). A total of 25 patients were randomized to receive placebo or pitrakinra (30 mg subcutaneously twice daily) for 28 days. The results of this trial have not yet been published (NCT00676884) [54].

### 2.5. IL-5 Directed Therapy

#### Mepolizumab

Mepolizumab is a fully humanized monoclonal antibody specifically directed against IL-5, the main factor of eosinophil growth, differentiation, and activation. It presents a high affinity and specificity against free IL-5, preventing it from binding to its receptor on the surface of eosinophils. Mepolizumab, therefore, decreases the number of eosinophils in blood, their tissular recruitment and the release of many pro-inflammatory mediators, causing tissue damage [55,56,57,58]. Although basophils also express such receptor, the mepolizumab inhibitory effect on them is lower because IL-3 is its main cytokine modulator [55].

An increased number of eosinophils in both peripheral blood and inflammatory infiltrate is a relatively frequent finding in AD [58]. Although eosinophils exact degree of involvement in the pathogenesis of AD is not well known, this fact confers to mepolizumab options as AD treatment.

A randomized, placebo controlled trial was carried out in several centers in Europe in 2002 in order to assess the efficacy of mepolizumab in the management of patients with AD (Table 1). No statistically significant differences were observed between both groups regarding the percentage of patients who achieved a 50% or greater clinical improvement on day +14 according to physician’s global assessment score. This difference became significant when any kind of improvement, like cut off point (> or <50%) was considered. Regarding blood eosinophils levels, a clear decrease was observed in patients treated with mepolizumab compared to placebo [59]. An excessively short treatment and the presence of other cytokines actively involved in eosinophil migration into sites of inflammation, such as eotaxin, could explain the lack of response to mepolizumab in these patients [56].

### 2.6. IL-12/23 Directed Therapy

#### Ustekinumab

Ustekinumab is a fully human monoclonal antibody capable of specifically binding to the p40 subunit of IL-12 and IL-23. It blocks both cytokine binding to their specific receptors on the lymphocyte surface. The result is the inhibition of differentiation and clonal expansion of naive cells into Th1 or Th17 responses respectively [60,61,62,63]. However, the involvement in the AD pathogenesis of IL-17 and IL-22, effector cytokines of Th17 cells, is not quite well known [63,64]. Batista *et al.* showed, comparing to controls, an increase of IL-17A levels both lesional skin and circulating in a study of 33 patients with AD, supporting the presence of a mixed inflammatory Th1/Th2/Th17 profile in the pathogenesis of AD [64]. High levels of IL-17 in acute skin lesions [65,66] and peripheral blood [66] from patients with AD were already shown. The potential role of IL-17A as a stimulating of Th2 response in acute phase of AD is more recent [63].

Fernández-Antón *et al.* published a few months ago, their experience with ustekinumab after treating four patients with severe AD refractory to oral corticosteroids, phototherapy and at least two systemic therapies (Table 1). In all cases, a significant clinical improvement was observed after the second or third dose of treatment [60]. A complete resolution of the lesions and pruritus was also observed in two cases of severe refractory DA, one month [62] and four months [61] after initiating ustekinumab treatment. In both cases, the improvement persisted 12 months after starting treatment. Currently, two phase II, RCDB trials are being conducted to evaluate the efficacy of ustekinumab in the treatment of severe AD. The first one is still recruiting patients. It consists of only two treatment arms, ustekinumab 45 mg *vs*. placebo (NCT01806662) [67]. The second one is being carried out in Japan. A total of 79 patients were randomized to placebo or different doses of ustekinumab (45 or 90 mg). Results are not yet available (NCT01945086) [68].

### 2.7. IL-22 Directed Therapy

IL-22 is a novel cytokine involved in the pathogenesis of AD. It contributes to the epidermal barrier dysfunction, and also seems to be responsible for the characteristic epidermal hyperplasia [10,12,69]. Nograles *et al.* demonstrated an elevation of IL-22 expression in AD lesional skin compared to normal and psoriatic skin biopsies [69]. Subsequently, a significant increase of this cytokine in both acute and chronic phases of AD was demonstrated by Gittler *et al.* [12].

Currently, the first study in patients with moderate to severe AD is in the recruitment process (NCT01941537) (Table 1) [70].

### 2.8. IL-31 Directed Therapy

The IL 31 is a very recently discovered cytokine mainly produced by Th2 and, in a lesser extent, by Th1. It seems to be involved in both acute and chronic phases of AD [10,71,72]. An increased expression of this cytokine on the inflammatory infiltrates in biopsies of patients with AD compared with normal skin and other inflammatory skin diseases has recently been confirmed by Nobbe *et al.* [73]. Previously, transgenic mice with overexpression of this interleukin had already been able to develop skin lesions similar to those of AD and intense pruritus [72]. In fact, administration of anti-IL-31 at doses of 10 mg/kg every five days for seven weeks in NC/Nga mice (an AD murine model) achieved a significant decrease in pruritus, although no effect on eczematous lesions was observed [71]. Subsequently, Kasutani *et al.* also supported the usefulness of these antibodies not only in the management of pruritus, but also in the management of skin lesions in AD-like murine models (BALB/c mice) [74]. Currently, the first two clinical trials with anti-IL-31 antibodies are ongoing (Table 1).

The first one is a phase I, single-dose, dose-escalation, RCDB trial in order to evaluate the safety and tolerability of the drug. Different subcutaneous and intravenous doses are going to be evaluated. It is still in recruitment process and there is no data available (NCT01614756) [75]. The second one is a phase II, multiple-dose and RCDB study. It is estimated that a total of 250 patients with AD will be recruited to evaluate the safety, tolerability and drug efficacy. Placebo or one of the four available doses will be assigned. No results are available either (NCT01986933) [76].

### 2.9. LFA-1 Directed Therapy

#### Efalizumab

Efalizumab is a humanized monoclonal antibody targeting the CD11a, a subunit of Lymphocyte function-associated antigen 1 (LFA-1) [77]. It prevents LFA-1 from binding to ICAM-1, an intercellular adhesion molecule expressed on the surface of endothelial and antigen presenting cells. The result is a reduction in T lymphocyte recruitment at the site of inflammation and a lower T-cell activation [78,79,80].

Efalizumab use in adult patients with moderate to severe plaque psoriasis was approved in October 2004 [77]. Later, its potential benefits in the management of AD began to be considered. Favorable results were obtained after administration of prophylactic anti-LFA-1 antibodies in 24 AD murine model (NC/Nga mice), corroborating the role of LFA-1 in the initial phases of AD [81]. However, since February 2009, efalizumab has no longer been authorized by the European Medicines Agency following three confirmed cases of progressive multifocal leukoencephalopathy [16].

Takiguchi *et al.* carried out a prospective study to assess efficacy and safety of 12 doses of efalizumab in patients with severe AD (Table 1). Promising results were observed, although they were not supported by a subsequent retrospective study conducted in Denmark (Table 1) [82].

### 2.10. LFA-3 Directed Therapy

#### Alefacept

Alefacept is a human dimeric fusion protein that blocks the interaction between LFA-3 and CD2, which are expressed on the surface of antigen-presenting cells and T lymphocytes respectively. Such interaction between LFA-3/CD2 during antigen presentation to T lymphocytes determined a costimulatory signal required for full T cell activation, a key point in the pathogenesis of AD [3,83,84]. It was approved by the U.S. Food and Drug Administration for treating moderate to severe psoriasis. However, its use was never approved in Europe and since 2011 it has not been available in the United States due to reasons beyond safety [3].

Moul *et al.* performed an open-label study in nine patients with moderate-severe AD (Table 1). A good clinical response was observed in only two patients. Overall, the treatment was well tolerated [85]. At the same time, a phase II study was performed in 10 patients with AD (Table 1). A significant clinical improvement was observed in all subjects and was maintained at least 10 weeks after treatment (NCT00376129) [84].

### 2.11. TNF-α Directed Therapy

The role of tumor necrosis factor alpha (TNF-α) as pro-inflammatory cytokine in the pathogenesis of multiple chronic inflammatory diseases is well known [5,86,87]. However, the use of TNF-α inhibitors in the management of AD is controversial. On one hand, there are only few publications where the use of etanercept and/or infliximab have demonstrated to be effective in the treatment of AD. On the other, a significant number of cases of anti TNF-α induced AD has already been published [88,89,90].

Etanercept is a human recombinant fusion protein capable of blocking the activity of TNF-α by preventing the binding to its receptor [86,91]. The number of cases of DA treated with etanercept is very scarce and the results are completely contradictory. Clinically significant improvement was observed in only two patients treated with 0.8 mg/kg of etanercept twice a week [91]. On the contrary, no improvement was observed in two patients in school age with a total weekly dose of 50 and 25 mg. In these cases, the treatment was discontinued at week 8 and 12 for lack of efficacy according to their EASI scores [86].

There is more experience in the use of infliximab for the management of AD, a chimeric monoclonal anti TNF-α. In a prospective study, a total of nine patients with moderate-severe AD underwent treatment with infliximab (Table 1). A clinically significant improvement was observed during the induction phase, with an average reduction of EASI and Pruritus Severity Assessment of 53% and 50% respectively at week two. Despite this promising initial response, only two of the nine patients maintained such clinical improvement until the end of follow up. In the remaining seven patients, the drug had been previously withdrawn at weeks 10, 14 and 30. The loss of efficacy was the reason for the withdrawal in six of the seven cases [87]. As the authors suggest, the use of infliximab in combination with low doses of other immunosuppressants could increase its efficacy, and prolong the therapeutic response. This methodology has already been used in other inflammatory diseases [87,92,93]. Cassano *et al.* also published their experience with infliximab after treating a 30-year-old patient with severe AD. Significant improvement of skin lesions and pruritus could be observed throughout the three years of follow-up [94].

We have not been able to find in the literature any case of AD successfully treated with adalimumab. A case of a 55-year-old male with AD and psoriasis treated with adalimumab has recently been published. Despite an initial worsening of AD, a continuous treatment with adalimumab may have obtained a good control of both diseases [95]. However, in our opinion the concomitant use of topical corticosteroids prevents assessing the true efficacy of adalimumab in the atopic eczema control.

### 2.12. TSLP Directed Therapy

Thymic stromal lymphopoietin (TSLP) is a novel cytokine involved in the pathogenesis of AD. Secreted by keratinocytes it promotes the activation of myeloid dendritic cells, favouring lymphocyte activation and a Th2-polarized response with the consequent release of pro-inflammatory cytokines [96]. A high expression of TSLP has been demonstrated in AD in both acute and chronic lesions [97,98]. More recently, however, a significant elevation of serum TSLP levels compared to unaffected subjects has been shown [99]. AMG 157 is a novel human monoclonal antibody that prevents the interaction of TSLP with its receptor. The first trial in patients with AD finished recently (Table 1). It was a phase I, RCDB study, in which a total of 78 patients were randomized to receive placebo or a single subcutaneous or intravenous dose of AMG 157. However, the results have not been published yet (NCT00757042) [100].

**Table 1:** Characteristics of three-or-more-patient studies included in the systematic review.

Drug Tested	Study Design	Dosage and Follow-Up	No. pts D/P		Average/Range and Sex	Results	Adverse Events	Current Role in AD Management
Alefacept [85]	Open-label	30 mg IM wkly (first 8 wks) + 30 mg or 15 mg wkly (following 8 wks) Follow-up: 48 wks	9	0	52y 6 M, 3 F	At wk 18: 2/9 pts ≥ EASI-50, 1/9 pts ≥ EASI- 90. 2/9 pts PGA-mild, 1/9 pts PGA-almost clear.	Well-tolerated. No serious AE. URI, sinus infection and herpes zoster.	Not further commercialized.
Alefacept [84]	Open-label	15 mg IM wkly (for 12 wks) Follow-up: 22 wks	10	0	19–51y 4 M, 6 F	10/10 significant improvement: Mean improvement of EASI: 78% at wk 12 and 86% at wk 22. Significantly decreased of pruritus and topical corticosteroids *p* < 0.001.	Well tolerated. No serious AE.	
Dupilumab (M4A) [48]	D-E, RCDB	75 mg SC wkly 150 mg SC wkly 300 mg SC wkly (for 4 wks) Follow-up: 4 wks	8 8 8	6	P: 37.4 ± 4.3 11 M, 5 F D: 42.6 ± 1.9 28 M, 23 F	At day 29: 19% (ppp) *vs*. 59% (ppd) ≥ EASI-50; *p* < 0.05. Mean improvement in the pruritus numerical rating scale: 18.6% (P) *vs*. 41.3% (D); *p* < 0.05.	Similar AE frequency in dupilumab and placebo groups. In relation to dupilumab: injection site reactions, nasopharyngitis and headache.	A marked and rapid clinical improvement was observed in the dupilumab group. Currently, it constitutes the main therapeutic promise in AD management. New clinical trials are currently being conducted which will provide more conclusive results [49,50,51].
Dupilumab (M4B) [48]	D-E, RCDB	150 mg SC wkly 300 mg SC wkly (for 4 wks) Follow-up: 4 wks	14 13	10				
Dupilumab (M12) [48]	P-G, RCDB	300 mg SC wkly (for 12 wks) Follow-up: 12 wks	55	54	P: 39.4 ± 1.7y 27 M, 27 F D: 33.7 ± 1.4y 31 M, 24 F	At day 85: 35% (ppp) *vs*. 85% (ppd) ≥ EASI-50; *p* < 0.001. Mean improvement in the pruritus numerical rating scale: 15.1% (P) *vs*. 55.7% (D); *p* < 0.001.		
Dupilumab (C4) [48]	P-G, RCDB	300 mg SC wkly plus topical GC (for 4 wks) Follow-up: 4 wks	21	10	P: 37.8 ± 5.3y 5 M, 5 F D: 36.0 ± 2.5 8 M, 13 F	At day 29: 50% (ppp) *vs*. 100% (ppd) ≥ EASI-50; *p* = 0.002. Mean improvement in the pruritus numerical rating scale: 24.7% (P) *vs*. 70.7% (D); *p* = 0.005.		
Efalizumab [79]	Open-label	Id of 0.7 mg/kg + 1 mg/kg wkly (for 12 wks). SC Follow-up: 20 wks	10	0	≥18y Unknown	At wk 12: 6/10 pts ≥ EASI-50, 2/10 pts ≥ EASI-75. Mean improvement of EASI: 52.3% (*p* < 0.0001). Mean decrease of VAS-pruritus of 2 cm (*p* < 0.015).	Well-tolerated. Secondary bacterial infections and viral infections. 1 case of thrombocytopenia.	Not further commercialized.
Efalizumab [82]	Case series	Id of 0.7 mg/kg + 1 mg/kg wkly (from 2 m to 12 m). SC Follow-up: unknown	11	0	40y/19–71y 3 M, 8 F	2/11 pts Improvement. 9/11 pts Drug withdrawal ≤ 6 m of beginning therapy. 7/11 pts Side effects.	Headache, secondary bacterial infection and lymphocytosis.	
Anti-IL-22 [70]	P-G, RCDB	600 mg IV at wk 0 + 300 mg IV at wk 2, 4, 6, 8, and 10 Follow-up: 20 wks	40 *	20 *	Unknown	No results are available. The study is ongoing.	Unknown.	No results are available. The first clinical trial is recruiting participants.
Anti Il-31 [75]	S-D, D-E, RCDB	0.01, 0.03, 0.06, 0.1, 0.3 or 1 mg/kg SC or 1, 3 or 10 mg/kg IV	96 *		Unknown	No results are available. The study is ongoing.	Unknown.	No results are available. The two first RCBD are being conducted.
Anti Il-31 [76]	D-E RCDB	Dosage not specified	264 *		Unknown	No results are available. The study is ongoing.	Unknown.	
Infliximab [87]	Open-label	5 mg/kg IV at wk 0, 2, 6, 14, 22, 30 and 38 Follow-up: 46 wks	9	0	19–60y 6 M, 3 F	At wk 2: 5/9 pts ≥ EASI-50. At wk 10: 4/9 pts ≥ EASI-50. Mean EASI 22.5 (wk 0), 10.6 (wk 2, *p* = 0.03), 15.1 (wk 10, *p* = 0.09). 2/9 Good response maintained.	Well-tolerated. Headache and nausea. 1 case of infusion-related reaction.	Clinical response tends not to be maintained over time, despite an initial good response.
Ligelizumab [38]	P-G, RCDB	Dosage not specified SC administration	22 **		Unknown	No results are available.	Previous studies in asthmatic pts showed headache, URI and injection site events [101].	Although the study has been completed, no study results have been published.
Mepolizumab [59]	P-G, RCDB	750 mg IV at wk 0 and 1 Follow-up: 30 days	20	23	29y/18–57y 20 M, 23 F	At day 14: 4.6% (ppp) *vs*. 22.2% (ppd) PGA score (0–2); *p* = 0.115. Mean SCORAD: 30.4 (P) *vs*. 29.0 (D); *p* = 0.293. Mean decrease of VAS-pruritus: 1.3 cm (P) *vs*. 2.6 cm (D); *p* > 0.05.	Well-tolerated. Fatigue and nausea.	The only RCBD performed in AD patients showed no clinical improvement. Scarce benefits in AD management.
Omalizumab [30]	Open label	150 mg SC or 300 mg SC every 2 or 4 wks Follow-up: 9 m	21	0	43y/14–64y 9 M, 12 F	21/21 pts statistically significant improvement in PGA score; *p* < 0.01. IgE baseline: 18.2–8396 IU/mL.	Well-tolerated. Injection site events and viral infections.	Although omalizumab has been shown to be effective in some case series, the only two RCDB performed showed no significant differences between the omalizumab and placebo group.
Omalizumab [31]	Case series	300 mg SC biwkly (8 cycles) Follow-up: 8 m	10	0	26.2y/19–35 y 6 M, 4 F	At wk16: Mean improvement of SCORAD: 25.2%. Mean decrease of VAS-pruritus of 2.6 cm (41.3%). At wk 24, 2/10 ≥ SCORAD-50. IgE baseline: 1704– >5000 IU/mL.	Well-tolerated. No signs of AE.	
Omalizumab [25]	Case series	450 mg SC every 3 wks 300 mg SC biwkly 600 mg SC every 3 wks (2–24 cycles) Follow-up: unknown	7 1 1	0	31y/26–42y 4 M, 5 F	8/9 pts Improvement (pruritus and quality of life). 2/9 pts Skin lesions improvement. IgE baseline: 1600–24,780 IU/mL.	Well-tolerated. 1 case of infusion-related reaction.	
Omalizumab [32]	Case series	150, 300 or 450 mg SC biwkly (12 cycles) Follow-up: 24 wks	3	0	11.6y/10–13y 2 M, 1 F	3/3 pts Significant improvement IgE baseline: 1990–6120 IU/mL	Well-tolerated. No signs of AE.	
Omalizumab [35]	Case series	450 mg SC biwkly (8 cycles) Follow-up: unknown	3	0	39.3y/34–48y 2 M, 1 F	At wk 16: 3/3 pts No improvement. IgE baseline: 5440–24,400 IU/mL.	Well-tolerated. No signs of AE.	
Omalizumab [33]	Case series	150 mg SC biwkly (10 cycles) Follow-up: 20 wks	11	0	37y/22–47y 7 M, 5 F	At wk 20: 2/11 pts SCORAD reduction of >50%. 4/11 pts SCORAD reduction of 25%–50%. 2/11 pts SCORAD increase of >25. IgE baseline: 1343–39,534 IU/mL.	Well-tolerated. No signs of AE.	
Omalizumab [34]	RCDB	0.016 mg/kg/IgE SC every 2 or 4 wks (for 16 wks) Follow-up: 20 wks	13	7	P: 26y/18–43y 1 M, 6 F D: 30y/18–47y 5 M, 8 F	No statistically significant difference in IGA and EASI. IgE baseline: 281.86 ng/mL (P)/372.78 ng/mL (D).	Well-tolerated Injection site reaction, vertigo and migraine.	
Omalizumab [37]	RCDB	150 -375 mg SC every 2–4 wks (for 24 wks) Follow-up: 30 m	4	4	11.6y/4–22y Unknown	No improvement. SCORAD reduction of 20%–50% (D) *vs*. 45%–80% (P). Mean IgE baseline: 218–1890 IU/mL.	Well-tolerated. No AE in relation to omalizumab.	
Pitrakinra [54]	P-G RCDB	30 mg SC twice daily	25 **		Unknown	No results are available.	Not specified.	Although the study has been completed, no study results have been yet published.
Rituximab [22]	Open-label	1000 mg IV wks 0 and 2 Follow-up: 24 wks	6	0	39y/19–63y 2 M, 4 F	At wk 4, 8, 16 and 22: 6/6 pts Significant decrease of EASI.	Well-tolerated. No severe AE. URI, otitis media, nausea and vomiting.	Promising results with doses used for RA. Only this 6-patient open-label study was performed. More studies will be necessary.
Anti-TSLP [100]	RCDB	Dosage not specified. SC and IV administration.	78 **		Unknown	No results are available.	No AE were observed in a previous study performed in asthmatic pts [102].	Although the study has been completed, no study results have been yet published.
Ustekinumab [60]	Case series	45 mg SC wk 0, 4 and every 12 wks (4–6 injections) Follow-up: 13 m	4	0	27y/23–29y 4 M	At wk 16: 4/4 pts SCORAD reduction of >50% (69.5, 73, 74.6 y 79% respectively). 4/4 pts VAS-pruritus reduction of >50% (70, 60, 60, 80% respectively).	Well-tolerated. No signs of AE.	The limited published reports showed very good results. Although the two first RCDB are being conducted currently and will provide accurate results [67,68].

AE, adverse events; Biwkly, biweekly (every two weeks); D, study drug group; D-E, dose-escalation; EASI, Eczema Area Severity Index; EASI-50, a 50% improvement in EASI score; F, female; Id, initial conditioning dose; GC, glucocorticoids; IGA, Investigator’s Global Assessment; IM, intramuscular; IV, intravenous; M, male; m, months; No., number; P, placebo group; P-G, parallel-group; PGA, Physician Global Assessment; ppd, percentage of patients in study drug group; ppp, percentage of patients in placebo group; PSA, Pruritus Severity Assessment; pts, patients; RA, rheumatoid arthritis; RCDB, randomized, controlled, double blind study; SC, subcutaneous; SCORAD, scoring atopic dermatitis; S-D, single-dose; URI, upper respiratory tract infection; VAS, visual analogue scale; wk, week; wkly, weekly; wks, weeks; y, years; * Number of patients estimated to be enrolled. The study is being conducted; ** Number of patients enrolled supposedly. No results have been yet published.

## 3. Conclusions

Many efforts have been made to establish an effective and safe alternative in patients with severe AD refractory to other systemic treatments. The occasional lack of response to conventional immunosuppressive therapy and the impossibility of long-term treatment have enhanced the need for a better and clearer understanding of the AD physiopathogenesis to find new therapeutic targets and possible drugs as potential effective and safe alternatives. Since the 1990s, when interferon gamma and intravenous immunoglobulins started to be used, the first benefits of biological therapy in the AD management have been observed. However, it was not until the last decade when the largest number of discoveries in this field took place. A great number of studies have recently been published and trialed; however, there is still a lack of homogeneous results and a good clinical response. The overlapping of most of the pathogenic pathways may be responsible for the limited effectiveness of some of these new drugs. It seems that dupilumab has been postulated as the main promising therapy. However, very recent biological drugs, such as anti-IL22, anti-IL-31 or pitrakinra, seem to be paving the way with no available results up to date. No definite recommendations can be given for the time being. More trials are needed to reach decisive conclusions. Costs, potential adverse reactions and, in most cases, a child target population often pose impediments to the progress in the study of these new drugs.
